# Robust osteogenic efficacy of 2α-heteroarylalkyl vitamin D analogue AH-1 in VDR (R270L) hereditary vitamin D-dependent rickets model rats

**DOI:** 10.1038/s41598-022-16819-7

**Published:** 2022-07-22

**Authors:** Miyu Nishikawa, Naruhiro Murose, Hiroki Mano, Kaori Yasuda, Yasuhiro Isogai, Atsushi Kittaka, Masashi Takano, Shinichi Ikushiro, Toshiyuki Sakaki

**Affiliations:** 1 Department of Biotechnology, Faculty of Engineering, Toyama Prefectural University, 5180 Kurokawa, Imizu, Toyama 939-0398 Japan; 2 Department of Pharmaceutical Engineering, Faculty of Engineering, Toyama Prefectural University, 5180 Kurokawa, Imizu, Toyama 939-0398 Japan; 3 Faculty of Pharmaceutical Sciences, Teikyo University, 2-11-1 Kaga, Itabashi, Tokyo 173-8605 Japan

**Keywords:** Drug discovery, Molecular biology, Diseases, Endocrinology

## Abstract

Active vitamin D form 1α,25-dihydroxtvitamin D_3_ (1,25(OH)_2_D_3_) plays pivotal roles in calcium homeostasis and osteogenesis via its transcription regulation effect via binding to vitamin D receptor (VDR). Mutated VDR often causes hereditary vitamin D-dependent rickets (VDDR) type II, and patients with VDDR-II are hardly responsive to physiological doses of 1,25(OH)D_3_. Current therapeutic approaches, including high doses of oral calcium and supraphysiologic doses of 1,25(OH)_2_D_3,_ have limited success and fail to improve the quality of life of affected patients. Thus, various vitamin D analogues have been developed as therapeutic options. In our previous study, we generated genetically modified rats with mutated Vdr(R270L), an ortholog of human VDR(R274L) isolated from the patients with VDDR-II. The significant reduced affinity toward 1,25(OH)_2_D_3_ of rat Vdr(R270L) enabled us to evaluate biological activities of exogenous VDR ligand without 1α-hydroxy group such as 25(OH)D_3_. In this study, 2α-[2-(tetrazol-2-yl)ethyl]-1α,25(OH)_2_D_3_ (AH-1) exerted much higher affinity for Vdr(R270L) in in vitro ligand binding assay than both 25(OH)D_3_ and 1,25(OH)_2_D_3_. A robust osteogenic activity of AH-1 was observed in *Vdr*(R270L) rats. Only a 40-fold lower dose of AH-1 than that of 25(OH)D_3_ was effective in ameliorating rickets symptoms in *Vdr*(R270L) rats. Therefore, AH-1 may be promising for the therapy of VDDR-II with VDR(R274L).

## Introduction

Vitamin D exerts various biological effects involved in calcemic, osteogenic, anti-cancer, and immune responses^1^. Vitamin D_3_, which is an animal vitamin D form, is sequentially converted to the active form, 1,25-dihydroxyvitamin D_3_ (1,25(OH)_2_D_3_), in the body. The initial C-25 hydroxylation of vitamin D_3_ by CYP2R1 and CYP27A1 occurs in the liver, and subsequent 1α-hydroxylation of 25-hydroxyvitamin D_3_ (25(OH)D_3_) by CYP27B1, which is a key enzyme in the production of active vitamin D, occurs in the kidneys^2^. Transcriptional activity of vitamin D via vitamin D receptor (VDR) is a classic molecular mechanism of vitamin D. VDR target genes often involve calcium homeostasis and osteogenesis. Once the VDR ligand binds to the VDR, heterodimerized VDR with 9-cis retinoid X receptor (RXR) is translocalized to vitamin D-responsive element (VDRE) of the target gene promoter^3^. As the binding affinity of 1,25(OH)_2_D_3_ for VDR is much higher than that of 25(OH)D_3_, impaired transcriptional activity of 1,25(OH)_2_D_3_ causes typical bone disorders such as osteomalacia and rickets associated with hypocalcemia.

Hereditary rickets can be divided into different types depending on amount of vitamin D in the plasma. Patients with vitamin D-dependent rickets type I (VDDR-I) show decreased plasma concentrations of 1,25(OH)_2_D_3_ due to CYP27B1 deficient mutation and successfully respond to 1,25(OH)_2_D_3_ treatment^4^. In contrast, vitamin D-dependent rickets type II (VDDR-II) patients caused by VDR null mutation show normal or elevated plasma 1,25(OH)_2_D_3_ and are resistant to physiologic dose of 1,25(OH)_2_D_3_^5,6^, which leads to impaired intestinal calcium absorption. Treatment of VDDR-II patients with high doses of oral calcium and supraphysiologic doses of 1,25(OH)_2_D_3_ had limited success. A more aggressive approach to improve the defect in intestinal calcium absorption is the long-term intravenous injection of calcium, which restores the serum calcium levels to normal and reverses rickets in some cases^7^. However, the excessive calcemic effect of natural 1,25(OH)_2_D_3_ causes risk factors such as hypercalcemia and ectopic calcification, while physiologic dose of 1,25(OH)_2_D_3_ is effective for hereditary VDDR-I patient. Therefore, various vitamin D analogues have been developed for the therapeutic options to eliminate the limitations of current therapeutic approaches. Hence, the in vivo evaluation system of exogenous VDR ligands might be a powerful method for the development of medical reagents including vitamin D analogues.

In our previous study, we generated genetically modified rats with a mutated Vdr(R270L). Rat Vdr mutation R270L is an ortholog of human VDR mutation R274L isolated from the VDDR type II rickets patient^6,8,9^, and exerts significant reduced affinity toward 1,25(OH)_2_D_3_ because rat Vdr Arg270 and human VDR Arg274 play a pivotal role in hydrogen bond-formation with the 1α-hydroxy group of 1,25(OH)_2_D_3_. Patients with VDR(R274L) are barely responsive to 1,25(OH)_2_D_3_. This VDR mutation does not affect affinity toward 25(OH)D_3_ because the VDR(R274L) protein maintains His305 and His397 residues, which are involved in hydrogen bonding with 25-hydroxy group of 25(OH)D_3_ and 1,25(OH)_2_D_3_^6,10^. In addition, “alopecia”, a typical symptom in VDR-null type rickets patients and animal models, was not observed in the *Vdr*(R270L) rats and human patients with VDR(R274L)^8,11^, suggesting that their VDR functions, such as DNA binding and hetero-dimer formation with RXR were normal. Thus, only the transcriptional action of 1,25(OH)_2_D_3_ via VDR can be significantly deficient in *Vdr*(R270L) rats, which can be used to evaluate biological activities of exogenous VDR ligand without 1α-hydroxy group, such as 25(OH)D_3_. In fact, *Vdr*(R270L) rats showed bone disorders associated with hypocalcemia and subsequent hyperparathyroidism even with significantly elevated plasma 1,25(OH)_2_D_3_ after weaning, indicating that 1,25(OH)_2_D_3_ cannot act as a high-affinity ligand of Vdr(R270L)^11^.

Previously, we failed to demonstrate the direct action of 25(OH)D_3_ via VDR in vivo using *Cyp27b1*-KO mice as Cyp27b1-independent generation of 1,25(OH)_2_D_3_ was observed in these mice with high doses of 25(OH)D_3_ treatment^12^. However, in in vivo studies using the *Vdr*(R270L) rats, we successfully demonstrated *Vdr*(R270L)-mediated action of 25(OH)D_3_, which is a precursor of 1,25(OH)D_3_ but acts as a weak VDR ligand^13–17^. Administration of high doses of 25(OH)D_3_ to *Vdr*(R270L) rats ameliorated rickets symptoms associated with decreased plasma 1,25(OH)_2_D_3_ to the normal level and increased plasma 25(OH)D_3_, strongly suggesting that the therapeutic effect of 25(OH)D_3_ is due to the direct binding of 25(OH)D_3_ to Vdr(R270L)^11^. This study showed that *Vdr*(R270L) rats are useful model for evaluating the exogenous Vdr ligands in vivo.

**Figure 1:**
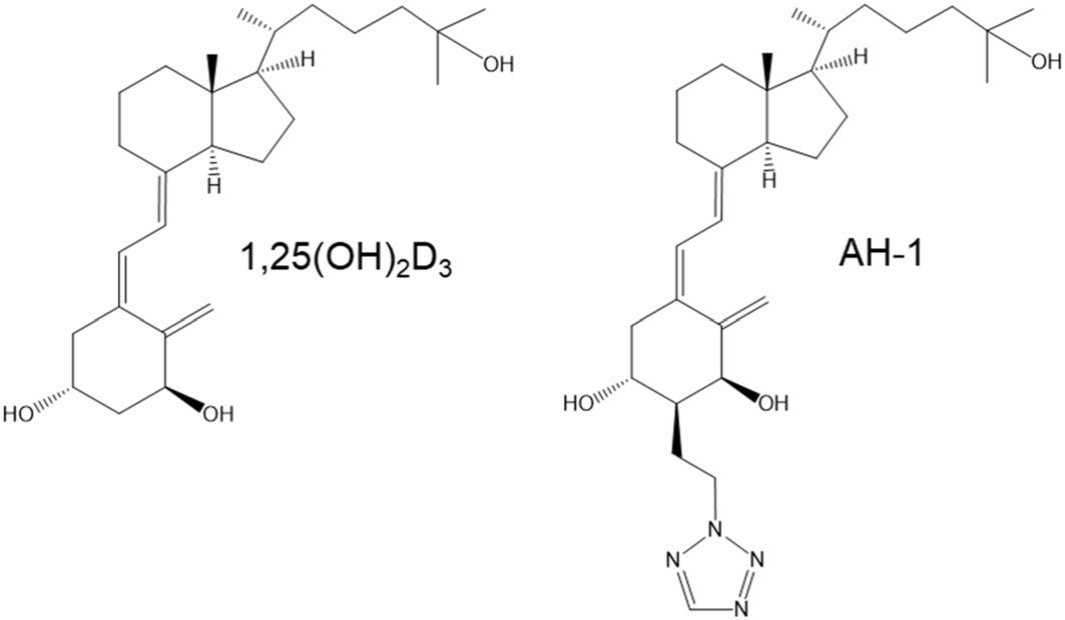
Chemical structures of 1,25(OH)_2_D_3_ and AH-1.

Here we show the osteogenic activity of the vitamin D analogue AH-1, in *Vdr*(R270L) rats. AH-1 is a C-2α-substituted vitamin D analogue with a heteroarylalkyl group (Fig. 1). AH-1 exhibits high osteocalcin promoter transactivation activity in human osteosarcoma cells^18^ and a robust osteogenic effect in ovariectomized (OVX) rats without hypercalcemia compared to 1,25(OH)_2_D_3_^19^. It was suggested that the additional hydrogen bond between the nitrogen of 2α-azole ring of AH-1 and Arg274 residue of VDR might strengthen the binding affinity, resulting in higher osteogenic efficacy^19^. In contrast, AH-1 also exhibited a 30 fold-higher affinity toward human VDR(R274L) than that of 1,25(OH)_2_D_3_ in binding assay using split-type luciferase anchored with truncated ligand-binding domain of human VDR(R274L), suggesting that AH-1 is also a potent agonist of rat Vdr(R270L)^20^. In this study, we evaluated the osteogenic effects of AH-1 on type II rickets model *Vdr*(R270L) rats.

## Results

### High binding affinity of AH-1 for Vdr(R270L) ligand binding domain

**Figure 2:**
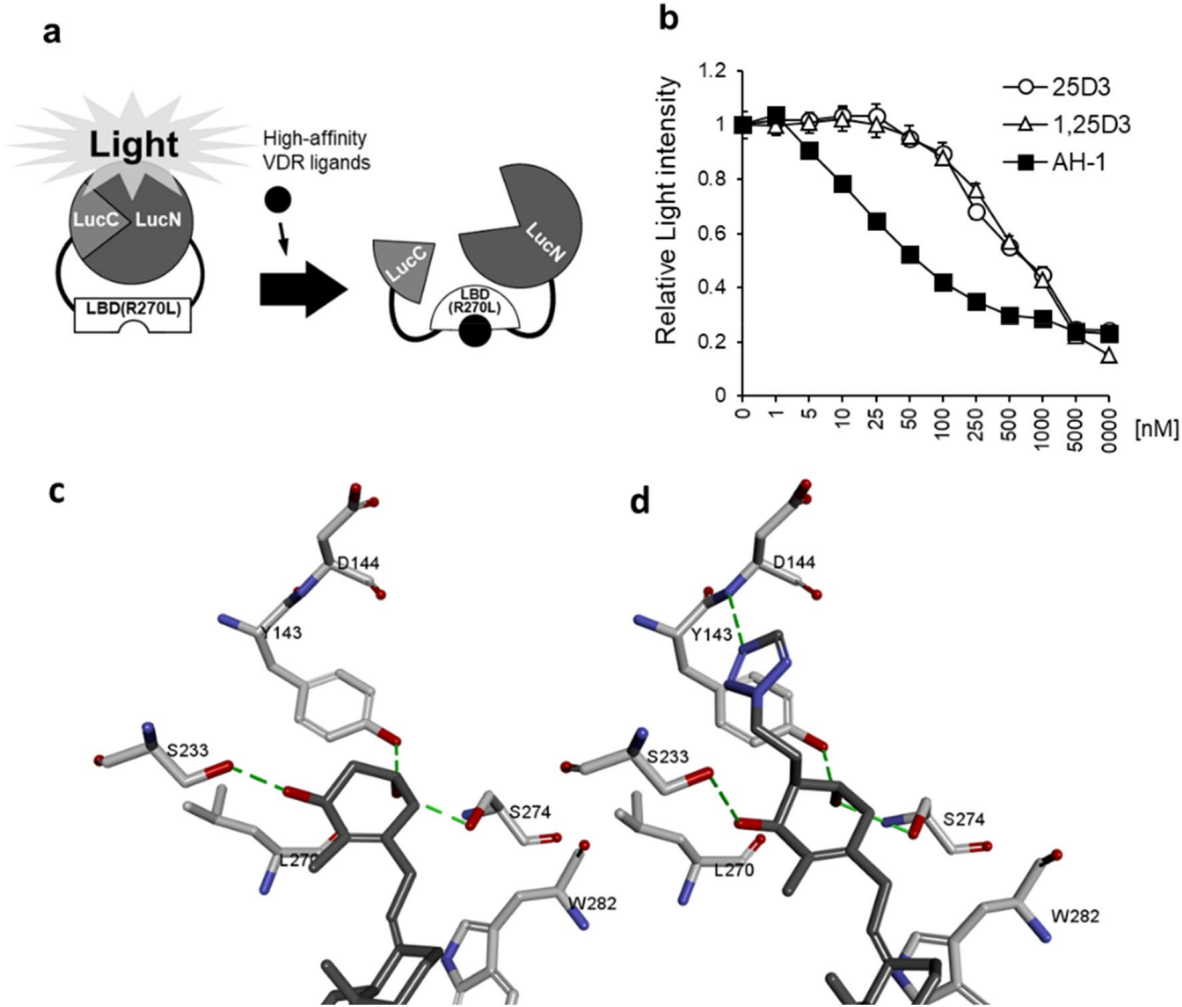
Interaction between AH-1 and Vdr(R270L). (**a**) Detection mechanism of the LucC-rat LBD(R270L)-LucN biosensor. Binding of the high-affinity Vdr ligand to the LucC-LBD(R270L)-LucN biosensor may cause a conformational change of LBD(R270L), and then disruption of the functional complex between LucN and LucC fragments of the split luciferase. (**b**) Comparison of binding affinity of 25(OH)D_3_, 1,25(OH)_2_D_3_ or AH-1 to LBD of rat Vdr. The relative light intensity compared to the control (0 nM = 1% EtOH) was shown. Data are represented as mean ± SEM (n = 3). (**c**) Complex structures of R270L mutant Vdr with 1,25(OH)_2_D_3_. (**d**) Complex structures of R270L mutant Vdr with AH-1, in which the A-ring parts are magnified. The carbon atoms are colored by grey for the ligands and by white for the amino-acid residues. Green dotted lines show hydrogen bonds.

In our previous study, we developed a bioluminescent sensor of VDR ligands consisting of split-luciferase and LBD of the human VDR^20–22^. Furthermore, we demonstrated that AH-1 exerted much higher binding affinity for human VDR(R274L) than 1,25(OH)_2_D_3_ and 25(OH)D_3_^20^. To confirm the binding affinity of AH-1 for rat Vdr(R270L), we examined the affinities of 25(OH)D_3_, 1,25(OH)_2_D_3_, and AH-1 for LBD of Vdr(R270L) (LBD(R270L)) using a bioluminescent sensor, in which light intensity is decreased when the ligand binds to LBD (Fig. 2a)^21,22^. As expected, the affinity of AH-1 for LBD(R270L) was much higher than those of 1,25(OH)_2_D_3_ and 25(OH)D_3_ (Fig. 2b).

### Prediction of interaction energies between Vdr(R270L) and AH-1

Computational docking was performed to construct a model of Vdr(R270L) complex with 1,25(OH)_2_D_3_ or AH-1, The complex structures demonstrated that Asp144 backbone N atom interacted with one of tetrazole ring nitrogen atoms of AH-1 at the distance of 2.5 Å (Fig. 2c,d). In addition, van der Waals interaction between tetrazole ring and ligand-free protein contributes stabilization of the complex. The binding energies of 1,25(OH)_2_D_3_ and AH-1 in the complex were calculated to be − 86.2 and − 106.7 (kcal/mol), respectively, suggesting that Vdr(R270L)-AH-1 complex is more stable than Vdr(R270L)-1,25(OH)_2_D_3_ complex (Supplemental Table 2).

### Therapeutic effect of AH-1 on growth and osteogenesis in *Vdr*(R270L) rats

**Figure 3:**
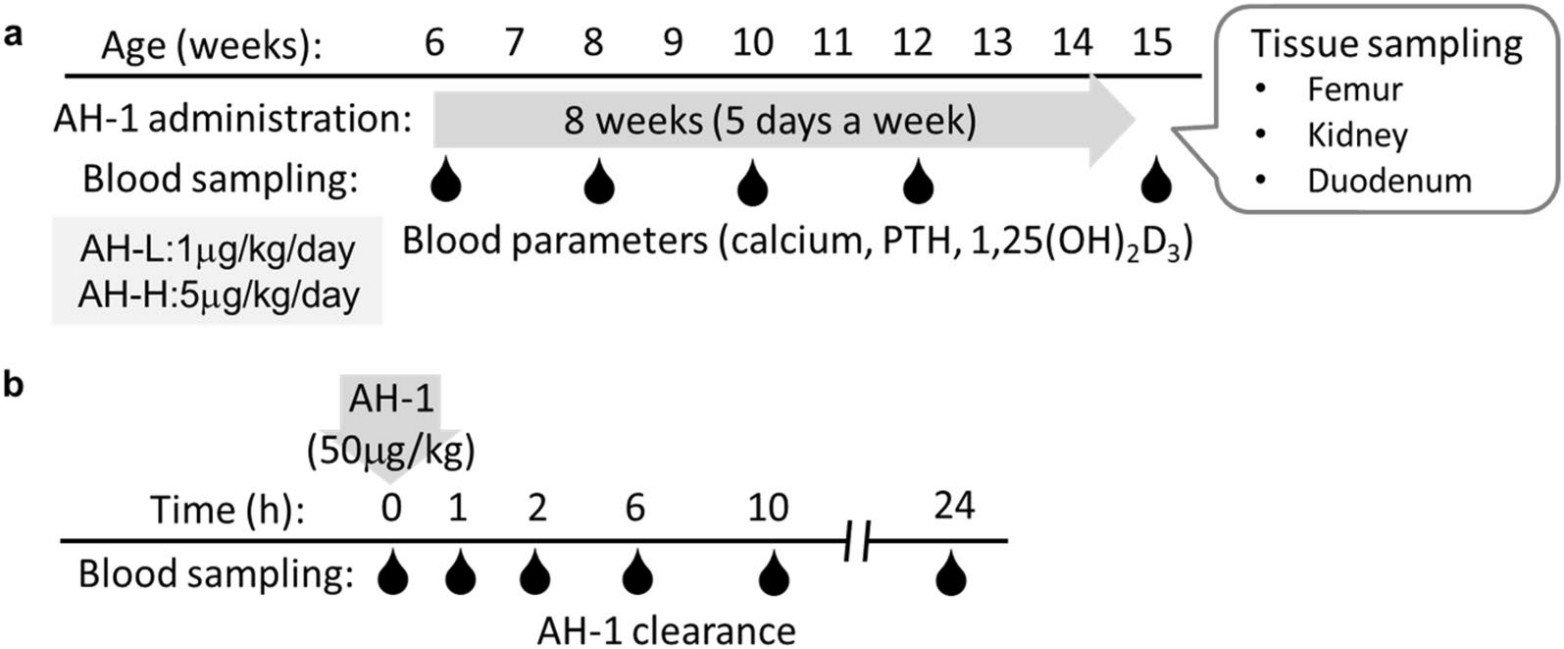
Experimental design of AH-1 treatment for Vdr(R270L) rats. (**a**) For long term treatment to evaluate osteogenic efficacy, AH-1 or vehicle was administrated from 6 to 15 weeks of age. Blood was collected at 6, 8, 10, 12 and 15 weeks old. Tissues were collected after 24 h of final dosing. (**b**) High dose of AH-1 was singly administrated for the AH-1 clearance analysis. The blood was collected from 0 to 24 h after the single administration.

**Figure 4:**
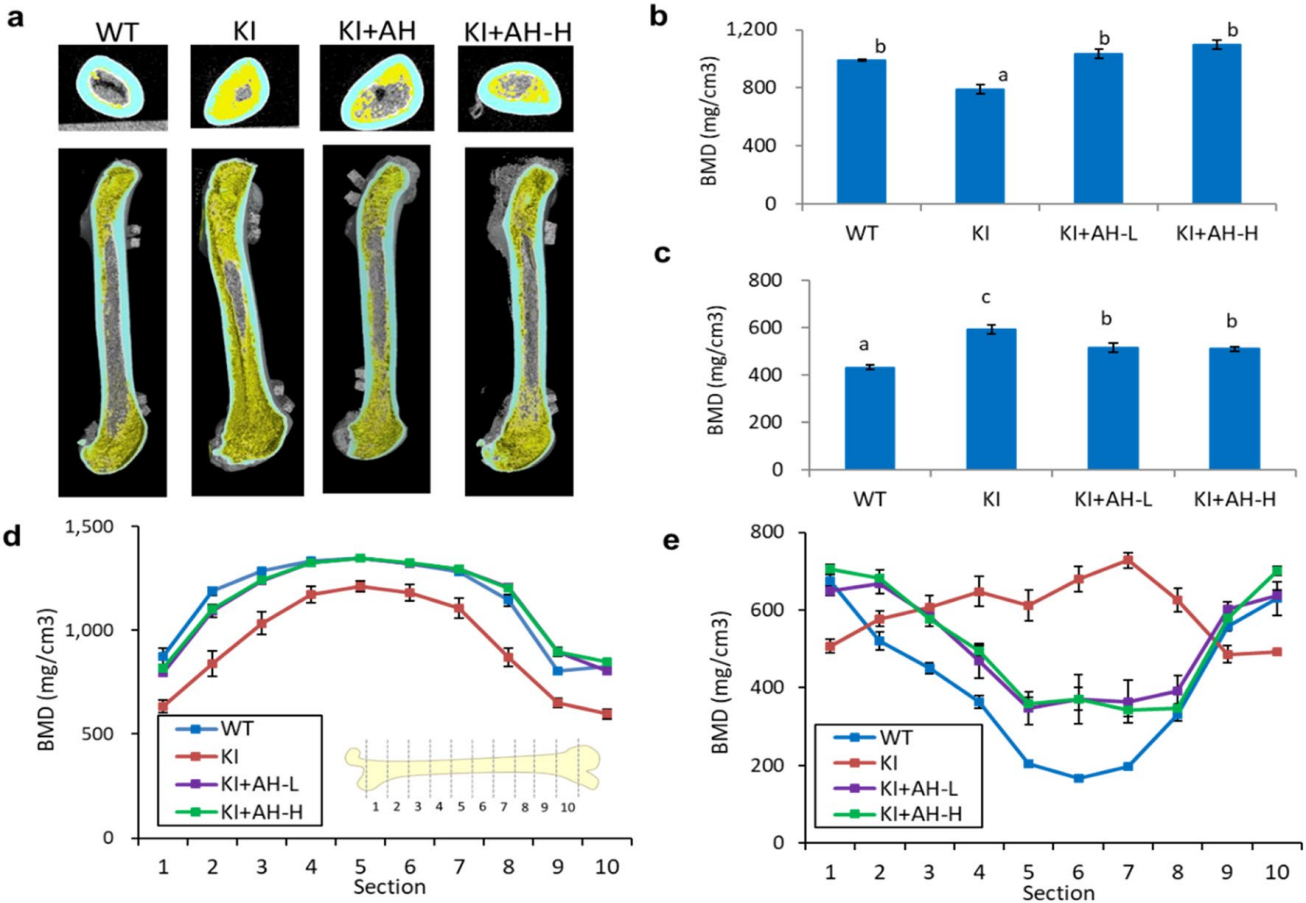
Impact of AH-1 on rickets bone phenotypes of Vdr(R270L) rats. (**a**) Femur structure with horizontal 2-D images at distal femur (upper panels) and vertical 3-D images of whole femur (lower panels) at 15 weeks old. Cortical and trabecular bone were colored with cyan and yellow, respectively. (**b**,**c**) Cortical (**b**) and trabecular (**c**) BMD of whole femur at 15 weeks old. Values of BMD are the means ± SEM (n = 5–8 animals/group). (**d**,**e**) Cortical (**d**) and trabecular (**e**) BMD distribution between horizonal 10 sections of whole femur at 15 weeks old. Section 1 and 10 are the most proximal and distal section, respectively. Values of BMD are the means ± SEM (n = 5 animals/group).

*Vdr*(R270L) rats at 6 weeks of age were orally administered with AH-1 five times a week for 8 weeks (Fig. 3). As previously reported^11^, *Vdr*(R270L) rats without AH-1 treatment showed bone disorders, including decreased bone mineral density (BMD) of cortical bones and hyperplasia of trabecular bones in the femur, which led to increased trabecular BMD and narrowed medullary cavity in femur (Fig. 4a–e). CT analysis revealed that AH-1 treatment ameliorated the abnormal morphological changes in the cortical and trabecular bones of *Vdr*(R270L) rat femur (Fig. 4a). Consistent with the effect of AH-1 on femur structure, AH-1 normalized cortical and trabecular BMD of *Vdr*(R270L) rat femurs in a dose dependent manner (Fig. 4b,c). In wild-type rat femurs, the cortical BMD was higher at the diaphysis than at the epiphysis, resulting in reverse U-shaped BMD distribution, as presented in Fig. 4d. While *Vdr*(R270L) rats showed decreased cortical BMD among all sections in femur, AH-1 treatment restored cortical BMD to the same levels as those of the wild-type rat femurs (Fig. 4d). The femur trabecular bone was highly distributed in the epiphysis in the wild-type rats, which showed a U-shaped curve when the trabecular BMD was plotted from proximal to distal along the major axis of the femur (Fig. 4e). The trabecular distribution in *Vdr*(R270L) rats was quite different from that in wild-type rats, showing a slightly reverse U-shaped curve (Fig. 4e). These distribution patterns are consistent with the 2D and 3D-CT images in Fig. 3a. AH-1 treatment partially normalized the distribution of trabecular BMD, which showed a normal U-shaped curve associated with a wide medullary cavity and increased cortical BMD (Fig. 4a,d,e). AH-1 treatment also increased the growth rate of Vdr(R270L) rats (Supplemental Fig. 1).

### Calcemic efficacy of AH-1 in *Vdr*(R270L) rats

**Figure 5:**
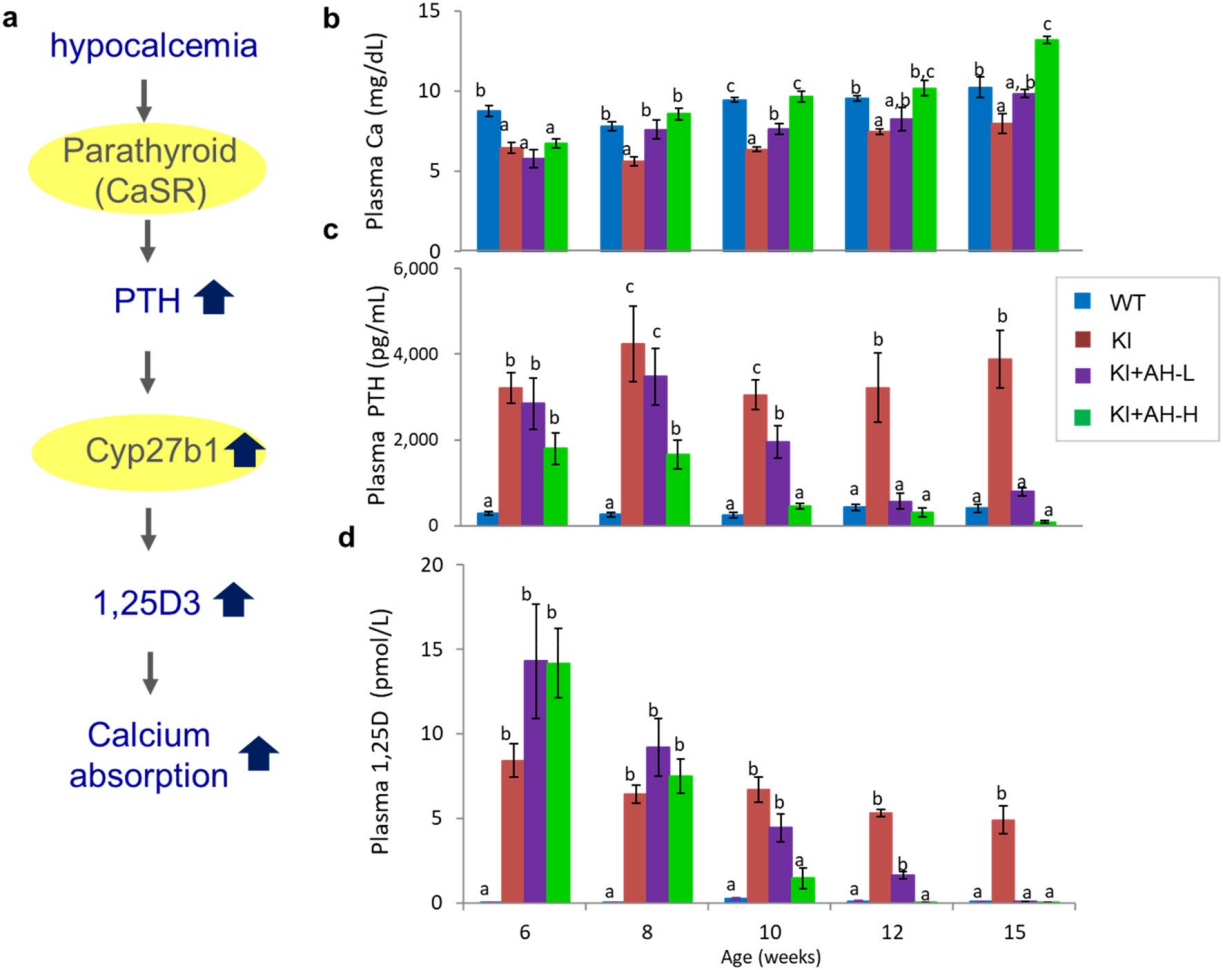
Calcemic effects of AH-1 in Vdr(R270L) rat plasma. (**a**) Feedback loop of plasma calcium, PTH and 1,25(OH)_2_D_3_. (**b**–**d**) Plasma calcium (**b**), PTH (**c**), and 1,25D (**d**) at 6,8,10, 12 and 15 weeks old. Values are the means ± SEM (n = 5–8 animals/group). Different letters: p < 0.05 by 2-way ANOVA.

The calcemic actions of 1,25(OH)_2_D_3_ via VDR are the most important molecular mechanisms underlying the osteogenic effect of vitamin D. We previously reported that the *Vdr*(R270L) rats at 15 weeks of age showed lower plasma calcium levels consistent with significant increased parathyroid hormone (PTH) and 1,25(OH)_2_D_3_, whose synthesis was triggered by the low plasma calcium level via calcium-sensing receptor (CaSR) in the parathyroid gland (Fig. 5a)^11^. *Vdr*(R270L) rats at 6 weeks of age already showed decreased plasma calcium associated with highly elevated PTH and 1,25(OH)_2_D_3_. In AH-H (5 μg/kg/time) group at 8 weeks of age, plasma calcium levels were fully normalized with subsequent normalization of PTH and 1,25(OH)_2_D_3_ level at 10 weeks of age (Fig. 5b–d). Plasma calcium parameters in the AH-L (1 μg/kg/time) group were also fully normalized at 15 weeks old (Fig. 5b–d). It was noted that these therapeutic effects of AH-1 were observed with reduced plasma 1,25(OH)_2_D_3_ to the normal level. These results suggest that not 1,25(OH)_2_D_3_ but AH-1 itself acts as a ligand of Vdr(R270L). In our previous study, we demonstrated that 25(OH)D_3_ acts as a weak ligand of Vdr(R270L) under high plasma levels of 25(OH)D_3_ (around 500 nM, which is more than 20-fold higher than the level in the wild-type) in *Vdr*(R270L) rats^11^. Hence, we also determined the plasma concentration of 25(OH)D_3_ by LC/MS/MS analysis. Plasma 25(OH)D_3_ levels in the *Vdr*(R270L) rats with or without AH-1 treatment were less than 20 nM, not significantly different from those in wild-type rats (Supplemental Table 3). These results suggest that 25(OH)D_3_ did not affect the calcium metabolism profiles of *Vdr*(R270L) rats.

### Transcriptional activities of AH-1 on Vdr target genes

**Figure 6:**
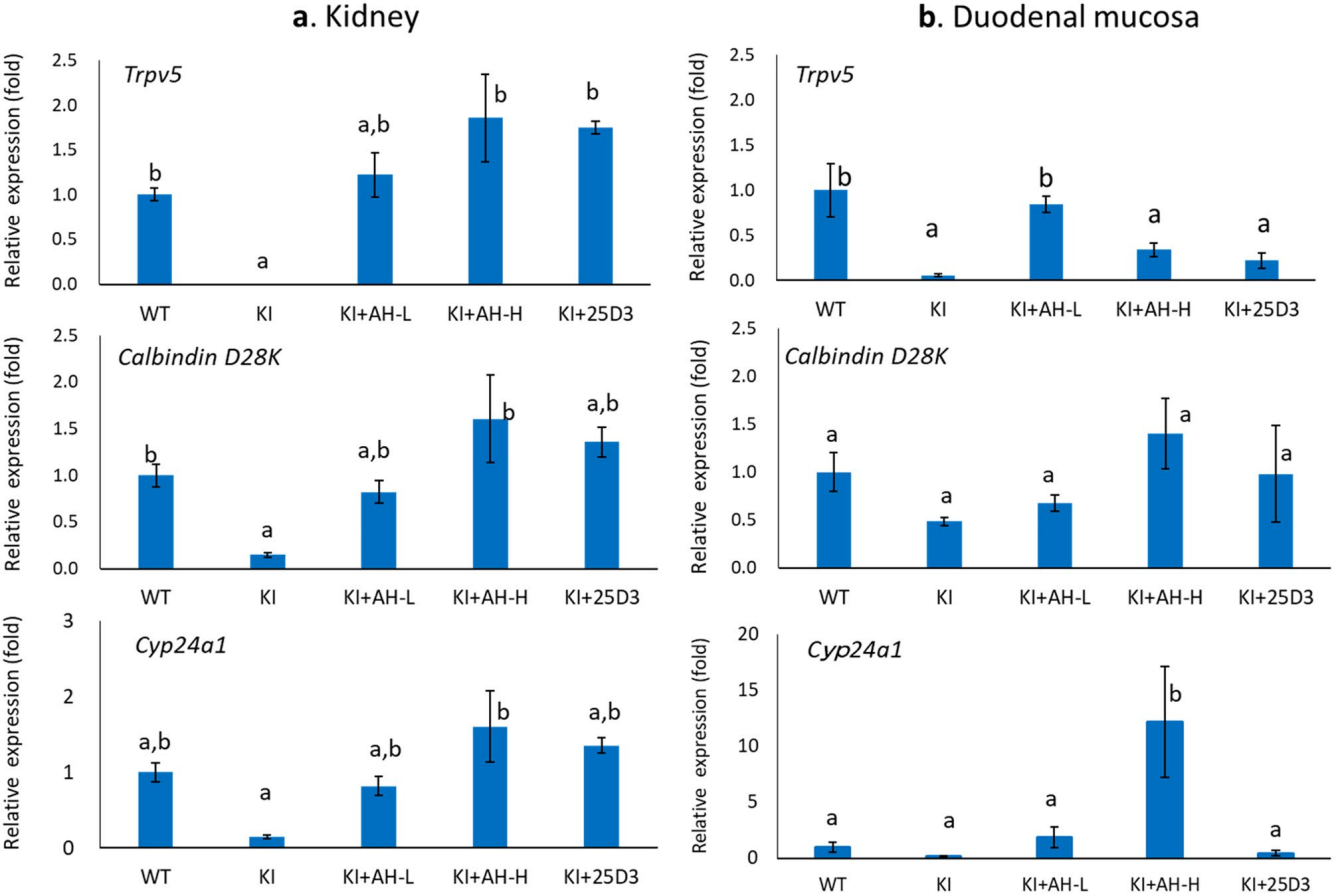
Transcriptional activity of AH-1 on Vdr target genes in intestine and kidney. Relative mRNA expression of *Trpv 5*, *Calbindin D28K* and *Cyp24a1* in kidney (**a**) and duodenal mucosa (**b**). Values are the means ± SEM (n = 4–8 animals/group). Different letters: p < 0.05 by 2-way ANOVA.

The duodenum and kidneys are important target organs of vitamin D, and are involved in vitamin D-dependent calcium absorption and reabsorption, respectively. Epithelial calcium transport is mediated by the calcium channels and intracellular calcium binding protein, such as calcium-selective transient receptor potential vanilloid subfamily member 6 (TRPV6) and calbindin-D9K in the intestine, or TRPV5 and Calbindin-D28K in the kidneys, both of which are vitamin D-responsive genes. Hence, we examined the transcriptional activity of AH-1 on these target genes in the duodenal mucosa and kidneys. The expression levels of renal *Trpv5* and *Calbindin D28K* were significantly lower in *Vdr*(R270L) rats, even though plasma level of 1,25(OH)_2_D_3_ was highly elevated (Figs. 5d and 6a). In contrast, the expression levels of these genes in the AH-1-treated group were elevated to the same levels as those in the wild-type rats, while plasma concentration of 1,25(OH)_2_D_3_ was decreased to the normal level, indicating that not 1,25(OH)_2_D_3_ but AH-1 enhanced Vdr(R270L)-dependent transcriptional activity (Figs. 5d and 6a). In addition, the expression level of duodenal *Trpv5* were extremely low in *Vdr*(R270L) rats and significantly induced in the AH-L group, whereas the expression levels of duodenal calbindin D28K was not significantly different between these groups (Fig. 6b). The transcriptional activity of AH-1 on these genes was compared to that of 25(OH)_3_ (200 μg/kg/day), the samples of which were prepared in our previous study^11^. The transcriptional activity at higher dose of AH-1 (AH-H, 5 μg/kg/day) was similar to that of 25(OH)D_3_ (200 μg/kg/day) (Fig. 6a,b).

CYP24A1, which is also a VDR target gene that is predominantly expressed in the kidneys, plays a pivotal role in vitamin D catabolism. Although basal expression level of *Cyp24a1* was suppressed in *Vdr*(R270L) rats, it was induced by AH-1 treatment in a dose-dependent manner (Fig. 6a,b). CYP24A1 mediates the sequential metabolism of the side chain of secosteroids, starting with hydroxylation at the C-24 or C-23 position, which is strongly involved in pharmacological efficacy of natural vitamin D and its analogues^23^. Hence, we examined plasma clearance of AH-1 in *Vdr*(R270L) rats after single dosing of 50 μg/kg AH-1 (Supplemental Methods 1 and 2), because the plasma levels of AH-1 were under detectable even in AH-H (5 μg/kg/time) group (data not shown). The plasma concentration of AH-1 in single-dose rats was approximately 20 nM after 24 h of dosing and decreased to 1.5 nM after 48 h (Supplemental Fig. 2). In our previous study, major metabolite of AH-1 by rat Cyp24a1 was identified as the 24*R*-hydroxylated form, and the *k*_cat_/*K*_m_ (μM^−1^ min^−1^) value of rat Cyp24a1 for AH-1 *24R*-hydroxylation was approximately 9% of that for 1,25(OH)_2_D_3_, suggesting that AH-1 is a poor substrate of rat Cyp24a1 compared with 1,25(OH)_2_D_3_^24,25^. Furthermore, 24*R*-hydroxylated AH-1 exhibited high binding affinity for VDR, which was 91% that of AH-1, and high HL-60 cell differentiation activity similar to AH-1^25^. Thus, we attempted to reveal Cyp24a1-mediated metabolism of AH-1 in *Vdr*(R270L) rats with a single dose of AH-1. Although 24*R*-hydroxylated AH-1 was detected slightly after 24 h of dosing, detailed metabolic pathway of AH-1 was not determined.

## Discussion

We demonstrated that AH-1 ameliorated the abnormal morphological changes of the femur associated with recovery from hypocalcemia in *Vdr*(R270L) VDDR-II rickets model rats (Figs. 4 and 5a). The highly elevated 1,25(OH)_2_D_3_ in *Vdr*(R270L) rats were reduced to the normal level in both the AH-L and AH-H groups (Fig. 5d). In addition, the expression of Vdr target genes, such as *Trpv5* and *Calbindin D28K*, was induced by AH-1 treatment (Figs. 6a,b), which promoted calcium absorption in the intestine and kidney. These results strongly suggest that AH-1 exerted strong calcemic effects by transcriptional action via binding to Vdr(R270L), which results in the normalization of femur structures. In fact, the split-luciferase-based ligand-binding assay demonstrated that AH-1 exerted a much higher binding affinity for Vdr(R270L) than 1,25(OH)_2_D_3_ and 25(OH)D_3_ (Fig. 1c). Based on these results, the therapeutic efficacy of AH-1 on *Vdr*(R270L) rat rickets symptoms could be attributed to the calcemic effect of AH-1 through its transcriptional activity mediated by its binding to Vdr(R270L).

In our previous study, we demonstrated that a high dose of 25(OH)D_3_ (200 μg/kg/day) normalized the rickets phenotypes of Vdr(R270L) rats, including the femur structure and plasma parameters for calcium metabolism. These results suggest that 25(OH)D_3_ could act as a physiologically essential ligand of Vdr(R270L) under conditions with higher plasma levels of 25(OH)D_3_ (around 500 nM, which is more than 20-fold higher than that in wildtypes)^11^. In the present study, plasma 25(OH)D_3_ levels in the AH-L and AH-H groups were not significantly different from those in the wild-type rats and were no more than 20 nM (Supplemental Table 3). Taken together, the biological effects of 25(OH)D_3_ on Vdr(R270L) were negligible in the AH-L and AH-H groups. It should be noted that the dose in the AH-H group was only 5 μg/kg/day, which was 40-fold lower than that of 25(OH)D_3_. Even a 1 μg/kg/day treatment (AH-L) might be effective when the treatment is started at a younger age. These results strongly suggest that AH-1 may be a potent therapeutic drug for Vdr(R270L) rats.

Originally, AH-1 was designed to exert enhanced binding affinity toward the wild-type VDR by forming a hydrogen bond between Arg274 of human VDR and a nitrogen atom of the heteroaromatic ring anchored with side chain at C-2α-position^19^. In fact, AH-1 at low doses of 0.007 and 0.02 μg/kg/day exerted high therapeutic effects for increasing BMD in OVX wild-type rats, which were approximately 3.5- and 5 -fold lower doses than the equivalent effective dose of 1,25(OH)_2_D_3_ (0.025 and 0.1 μg/kg/day)^19^. Arg274 of human VDR, which corresponds to Arg270 of rat VDR, plays a pivotal role in anchoring the 1α-hydroxy group of 1,25(OH)_2_D_3_. Thus, we assumed that the substitution of these Arg residues with leucine may cause a drastic decrease in the affinity toward not only 1,25(OH)_2_D_3_^6,26^ but also AH-1 because the additional hydrogen bond between AH-1 and Arg274 may be lost. However, a split-luciferase-based binding assay revealed that AH-1 maintained a high affinity for human VDR(R274L), whereas the affinity of 1,25(OH)_2_D_3_ for the VDR(R274L) significantly decreased^20^. These results led us to evaluate the therapeutic efficacy of AH-1 in rickets in *Vdr*(R270L) rats. As expected, AH-1 treatment normalized rickets phenotypes, including hypocalcemia and osteodysplasia by reducing plasma PTH and 1,25(OH)_2_D_3_ to normal levels.

The unexpected high potency of AH-1 as an agonist for human VDR(R274L) and rat Vdr(R270L) raises the question that how AH-1 interacts with the LBD of mutant VDRs lacking Arg274 or Arg270. The computational docking approach between Vdr(R270L) and the ligands demonstrated that Asp144 formed an additional hydrogen bond with the tetrazole nitrogen atom of AH-1 (Fig. 2), which led to a higher stability of the Vdr(R270L)-AH-1 complex than Vdr(R270L)-1,25(OH)_2_D_3_ complex. Thus, AH-1 could be a powerful agent for alternative therapeutic agent for rickets patients with VDR(R274L) mutation, as supraphysiological doses of current vitamin D-dependent therapeutic approaches such as 1,25(OH)_2_D_3_ treatment, have limited success^5,6^. In addition, *Vdr*(R270L) rats might be useful in vivo tool to evaluate the osteogenic efficacies of exogenous Vdr ligands.

In addition to AH-1, C2-substitution of natural vitamin D_3_ with a side chain is a potential approach for development of vitamin D agents for bone disorders^19,20,27–31^. In our previous study, five of 32 vitamin D analogues including AH-1 exhibited higher affinities for human VDR(R274L)-LBD than 1,25(OH)_2_D_3_. The four analogues, other than AH-1, were 2α-[-2-(1,2,4-triazol-1-yl) ethyl]-1α,25(OH)_2_D_3_ (MM-2)^19^, 2α-(3-hydroxypropoxy)-1α,25(OH)_2_D_3_ (O2C3)^27,28^, 2α-propoxy-1α,25(OH)_2_D_3_ (C3O1)^29^ and 2β-(3-hydroxypropoxy)-1α,25(OH)_2_D_3_ (ED-71)^30,31^. Among 5 analogues, AH-1 and MM-2 exerted the highest affinity for VDR(R274L)^20^, suggesting that the azole ring plays crucial role in the formation of a stable complex with human VDR(R274L) or rat Vdr(270L).

As shown in Fig. 6, administration of AH-1 (1 or 5 μg/kg/time) five times a week significantly induced the expression of *Cyp24a1* which is responsible for the metabolism of vitamin D analogues to determine their pharmacological efficacy. Clearance analysis after single dosing with a high dose of AH-1 (50 μg/kg) revealed that the plasma concentration of AH-1 after 24 h of dosing was approximately 20 nM, which is 35% of maximum plasma concentration (Supplementary Fig. 2). Although we tried to detect Cyp24a1-mediated metabolites in *Vdr*(R270L) rats with a single dose of AH-1, its 24*R*-hydroxylated product was only slightly detected after 24 h of dosing (Supplementary Fig. 2). The plasma concentration curve of AH-1 showed a bimodal curve, suggesting that a conjugation reaction and enterohepatic circulation may be involved in the metabolism of AH-1 (Supplementary Fig. 2). Further pharmacokinetic analyses are required to reveal the metabolism of AH-1 in Vdr(270L) rats.

Novel mechanisms of vitamin D has been also reported in addition to the classic molecular mechanism of vitamin D. They include VDR-independent action of 1,25(OH)_2_D_3_ and ligand independent action of VDR^8,32–35^. We and Asano’s group have also reported that direct actions of 25(OH)D_3_ in VDR-dependent or independent manner, respectively^11,36^. More recently, alternative nuclear receptors such as retinoid-related orphan receptors (RORs), aryl-hydrocarbon receptor (AhRs), and liver X receptors (LXRs) were reported as target of the hydroxyderivatives of vitamin D, which include 1,25(OH)_2_D_3_^37–39^. These alternative pathways of vitamin D signaling to be also considered for the elucidation of molecular mechanisms of the vitamin D action including its analogues.

In summary, we successfully demonstrated the therapeutic effects of synthetic VDR ligand, AH-1, in *Vdr*(R270L) rickets model rats. AH-1 may be a potent agent for treating VDDR-II patients with VDR(R274L). In addition, *Vdr*(R270L) rats may be useful for evaluating the response to exogenous natural hormones or synthetic VDR ligands in vivo.

## Materials and methods

### Materials

AH-1 was synthesized as previously described^19^. D6-25-hydroxyvitamin D_3_ (26,26,26,27,27,27-D6, d6-25(OH)D_3_) and LCMS grade organic solvents were purchased from Sigma-Aldrich (St. Louis, MO, USA). 4-[2-(6,7-dimethoxy-4-methyl-3-oxo-3,4-dihydroquinoxalyl)ethyl]-1,2,4-triazoline-3,5-dione (DMEQ-TAD) was purchased from FUJIFILM Wako Pure Chemicals (Osaka, Japan). Authentic standards of 24R,25(OH)_2_D_3_, and 24-oxo-25(OH)D_3_ were prepared as previously described^25^. Other chemicals were commercially available and of the highest quality.

### In vitro luciferase complementation assay

LucC-rat LBD(R270L)-LucN biosensor proteins were constructed and expressed as described in our previous reports^20–22^. The *E.coli* lysates containing the LucC-rat LBD(R270L)-LucN proteins were used for in vitro luciferase complementation assay.

The lysates were diluted with reaction solution [25 mM Tris–HCl (pH 7.4), 2 mM DTT, 0.2 mg/ml BSA] and the total volume was adjusted to the 50 μl. The threefold dilution of LucC-rat LBD(R270L)-LucN biosensor was plated into 96 well plate. Next, 25(OH)D_3_, 1,25(OH)_2_D_3_, or AH-1 in ethanol was added to the well at 0–10,000 nM and incubated at room temperature (23–26 ℃) for 30 min (pre-incubation). Then, 50 μl of luciferin solution [25 mM Tris–HCl (pH 7.4), 20 mM MgSO_4_, 2 mM d-Luciferin (Thermo Scientific, CA, USA) and 4 mM ATP] was injected into 96 well plate. At 30 min after addition of luciferin solution, the light intensity was measured by a luminometer (Infinite 200 Pro M Plex, TECAN). In this study, the relative light intensity in the presence of the 25(OH)D_3_, 1,25(OH)_2_D_3_ or AH-1 was calculated in comparison with the light intensity in the absence of the ligands (0 nM = 1% EtOH).

### Computational protein–ligand docking between Vdr(R270L) and ligands

The models were constructed based on the X-ray crystallographic complex structure of the mutant protein (pdb ID: 3VT3)^26^ with 1,25(OH)_2_D_3_ as follows. The native ligand conformation was in situ minimized at the binding site of VDR in the complex structure with CHARMm force field^40^. To model the interaction between Vdr(R270L) and AH-1, docking models of AH-1 and the ligand-free Vdr(R270L) were obtained with CDOCKER simulation^41^. The conformation of the best docking pose was in situ minimized with a similar method mentioned above. The binding energies were calculated with the finally obtained complex structures and were listed in Supplementary Table 2.

### Animals and diets

*Vdr*(R270L) rats were generated by CRISPR-Cas9 genome editing system as previously described^11^ and heterozygotes were bred to each other to obtain homozygotes and wildtypes. The rats were kept at controlled room temperature (22–26 °C), and in 50–55% humidity with a 12 h light/dark cycle. They were allowed food and water ad libitum and fed F-2 formula diet containing 0.75% Ca and 2000 IU vitamin D/kg diet^11^.

Male *Vdr*(R270L) rats were divided to three groups; vehicle control (KI), low dose of AH-1 (KI + AH-L, 1 μg/kg/day) and high dose of AH-1 (KI + AH-H, 5 μg/kg/day). AH-1 stock solution in EtOH was dissolved in corn oil for AH-1 groups. The equivalent volume of EtOH was dissolved in corn oil for vehicle control groups of wild type and *Vdr*(R270L) mutant. The reagents were orally administrated to the rats with 5 times a week from 6 to 15 weeks of age. Blood was collected from jugular vein to heparin-filled syringe under anesthetic condition with isoflurane at following endpoints of age; 6, 8, 10, 12 and 15 weeks old. Blood was centrifuged at 3000*g* for 10 min to obtain plasma. The plasma samples were stored at − 80 °C until subsequent experiments (Fig. 3a).

Twenty-four hours after the final administration, the rats were sacrificed under the anesthetic condition with isoflurane and tissues were collected after the blood collection. Duodenal mucosa and pieces of renal cortex were immediately soaked in ISOGEN II (Nippon Gene Co., Ltd, Tokyo, Japan) and homogenized, and then stored at − 80 °C until subsequent experiments. Femur was soaked in 70% EtOH and stored at 4 °C.

To examine the plasma clearance of AH-1, 50 μg/kg of AH-1 was administered to *Vdr*(R270L) rats at 15 weeks of age. After the single dosing, blood was collected at following endpoints; 0, 1, 2, 6, 12, 24 h after the dosing (Fig. 3b).

All experimental protocols using animals were performed in accordance with the Guidelines for Animal Experiments at Toyama Prefectural University and were approved by the Animal Research and Ethics Committee of Toyama Prefectural University.

### Computed tomography

In order to examine the morphological properties and BMD, micro computed tomography (μCT) analyses were carried out as previously described^11^. Briefly, the femurs were scanned by X-ray CT (Latheta LCT-200; Hitachi Aloka Medical, Tokyo, Japan) with a voltage of 50 kVp, a current of 500 μA, an integration time of 3.6 ms and an axial field of view of 48 mm, with an isotropic voxel size of 48 μm. The mineral content of the femur was calculated using LaTheta software (Hitachi Aloka Medical). A threshold density of 160 mg/cm^3^ was selected to distinguish mineralized from unmineralized tissue. The 3D-images of femur was constructed from the scanned images using VGSTUDIO 3.2 software (Volume Graphics, Heidelberg, Germany).

### Measurement of calcium metabolism parameters in plasma

The plasma calcium concentrations were measured using the Calcium E-Test Wako (FUJIFILM Wako Wako Pure Chemical, Osaka, Japan). The plasma parathyroid hormone (PTH) concentration was determined using the Rat Intact PTH ELISA Kit (Immutopics Inc., San Clemente, CA, USA). Plasma concentration of 1,25(OH)_2_D_3_ was measured using 1,25-(OH)_2_ Vitamin D ELISA Kit (Immundiagnostik, Bensheim, Germany) as described previously^11^.

### Real-time quantitative PCR

Total RNA was isolated from duodenal mucosa and renal cortex using ISOGEN II (Nippon Gene, Tokyo, Japan). cDNA was synthesized using PrimeScript RT Master Mix (Perfect Real Time) (TaKaRa, Otsu, Japan). Real-time PCR was carried out with Applied Biosystems 7500 Real-Time PCR System, by using TB Green TB Green Premix Ex Taq II (TaKaRa, Otsu, Japan) for the reaction reagent. The mRNA expression of *Cyp24a1*, *Trpv5* and *Calbindin D28k* were determined by the ΔΔCt method using *β-actin* as an internal control (Supplementary Table 1).

### Statistical analysis

The analysis was conducted with the use of IBM SPSS Statics software (version 25). Two-way ANOVA was performed for the analysis of the bone mineral density, length of femur, plasma calcium, plasma PTH, plasma 1,25(OH)_2_D_3_, and the mRNA expressions. Differences were considered significant at p < 0.05 by Bonferroni test.

### Ethics policy

This study was reported in accordance with ARRIVE guidelines (https://arriveguidelines.org).

## Supplementary Information

Supplementary Information.

## Data Availability

The datasets generated or analyzed during the current study are available from the corresponding author (T.S.) on reasonable request. A genomic sequence containing the mutated position is available in DDBJ data base accession number LC711029 (http://getentry.ddbj.nig.ac.jp/top-j.html).
