# Supplementary material for: Robust osteogenic efficacy of 2α-heteroarylalkyl vitamin D analogue AH-1 in VDR (R270L) hereditary vitamin D-dependent rickets model rats
Source: Sci Rep. 2022 Jul 22;12:12517. doi: 10.1038/s41598-022-16819-7 (PMC9307643; doi:10.1038/s41598-022-16819-7)
Supplement: Supplementary file 1 — Supplementary Information. [file 41598_2022_16819_MOESM1_ESM.pdf]

Supplemental Method

1. Single dosing of AH-1 for plasma clearance study.

Vdr(R270L) rats were orally administrated with 50 µg/kg of AH-1. Blood was collected from jugular vein at 1, 2, 6, 12, 24 and 48h after dosing. The blood containing heparin was centrifuged at 3,000 g for 10 min to collect plasma. The resultant plasma samples were stored at -80 °C until subsequent analysis.

2. LC/MS/MS analysis of AH-1 in the plasma.

Plasma concentrations of AH-1 were determined by using a modified method of LC-APCI-MS/MS [11]. Briefly, protein precipitation was performed by using acetonitrile with d6-25(OH)D_3 as an internal standard. The supernatant was evaporated and the residue was dissolved with 400 µL of ethyl acetate and 200 µL distilled water to extract the target compounds. After vigorously shaking and following centrifugation, ethyl acetate phase was taken and evaporated. After that, AH-1 and endogenous vitamin D metabolites were derivatized by DMEQ-TAD [34]. Separation was carried out using a reverse-phase C18 analytical column (CAPCELL PAK C18 UG120, 5µm; (4.6 I.D. × 250 mm) (SHISEIDO, Tokyo, Japan) with a solvent system consisting of (A) acetonitrile, (B) H2O (0-5 min A=30%, 5-34 min (A)=30→70 %, 34-37 min min (A)=70→100 %) as the mobile phase and a flow rate of 1.0 mL/min. All MS data were collected in the positive ion mode and quantitative analysis was carried out using MS/MS-MRM of the precursor/product ion for DMEQ-TAD-AH-1 (m/z : 847.3/564.1), DMEQ-TAD-24-OH-AH-1 (m/z：874.3/564.1) with a dwell time of 200 ms.

Supplemental Table1 Primer information used for qPCR analysis.

Target genes	GeneBank Accession No.	Primer sequences (5' to 3')	
Calbindin D28K	NM_031984	forward	tatgacactgaccacagtg
		reverse	aggtctgtgtactcagcaag
Trpv5	NM_053787	forward	cagacatcttcagagttggtgcct
		reverse	catcaccataatcagtagcacaaggg
Cyp24a1	NM_201635	forward	agcccggggcagatttcctctg
		reverse	catattcctcaggtcttccgc
β-actin	NM_031144	forward	aggcccagagcaagagaggcat
		reverse	ccatatcgtcccagttggtgaca

Supplemental Table2 Binding energies of native and synthetic ligands in the complex structures of Vdr(R270L).

VDR	$1,25(OH)_2D_3$[1]	AH-1[2]
R270L	-86.2<br>(kcal/mol)	-106.7<br>(kcal/mol)

[1]The binding energies of $1,25(OH)_2D_3$ were calculated by the *in situ* ligand minimization based on the X-ray crystallographic structures of the complexes with the mutant VDR (3VT3).
[2]The binding energies of AH-1 were calculated by the *in situ* ligand minimization based on the docking models with the apo forms of the mutant VDR (3VT3).

Supplemental Table3 Plasma 25(OH)D_3 concentration of wildtype (WT) and *Vdr*(R270L) (KI) rats at 15 weeks old.

Group	Plasma 25(OH)D_3 (nM)
WT	19.9±1.4
KI	15.5±1.2
KI+AH-L	17.6±3.4
KI+AH-H	11.7±1.0

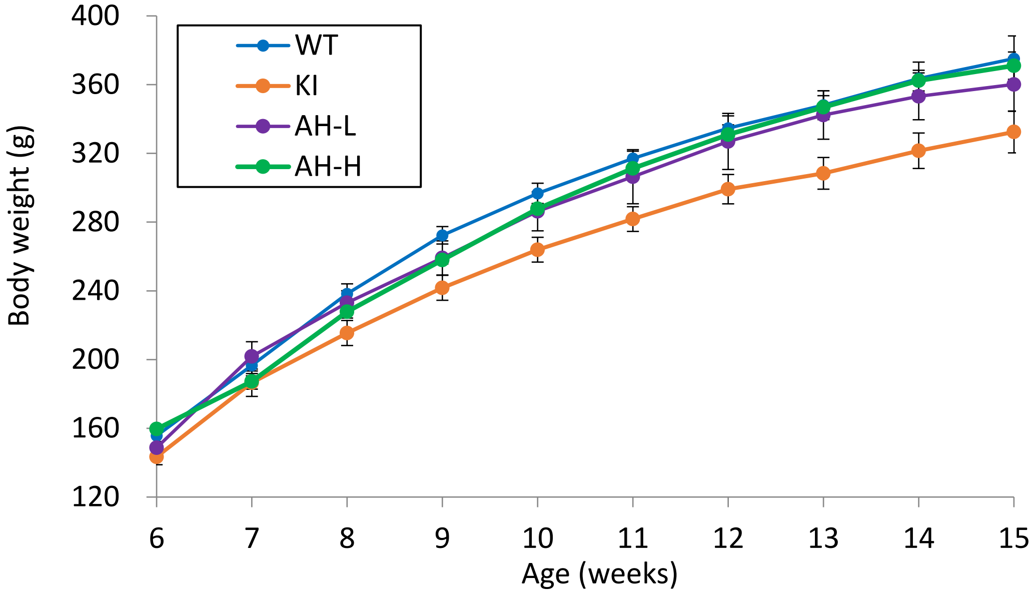

Supplemental Figure 1 Effect of AH-1 on growth of Vdr(R270L) rats.
Growth curve of body weight from 6 to 15 weeks old. Values are the means ± SEM (n=5-7 animals/group).

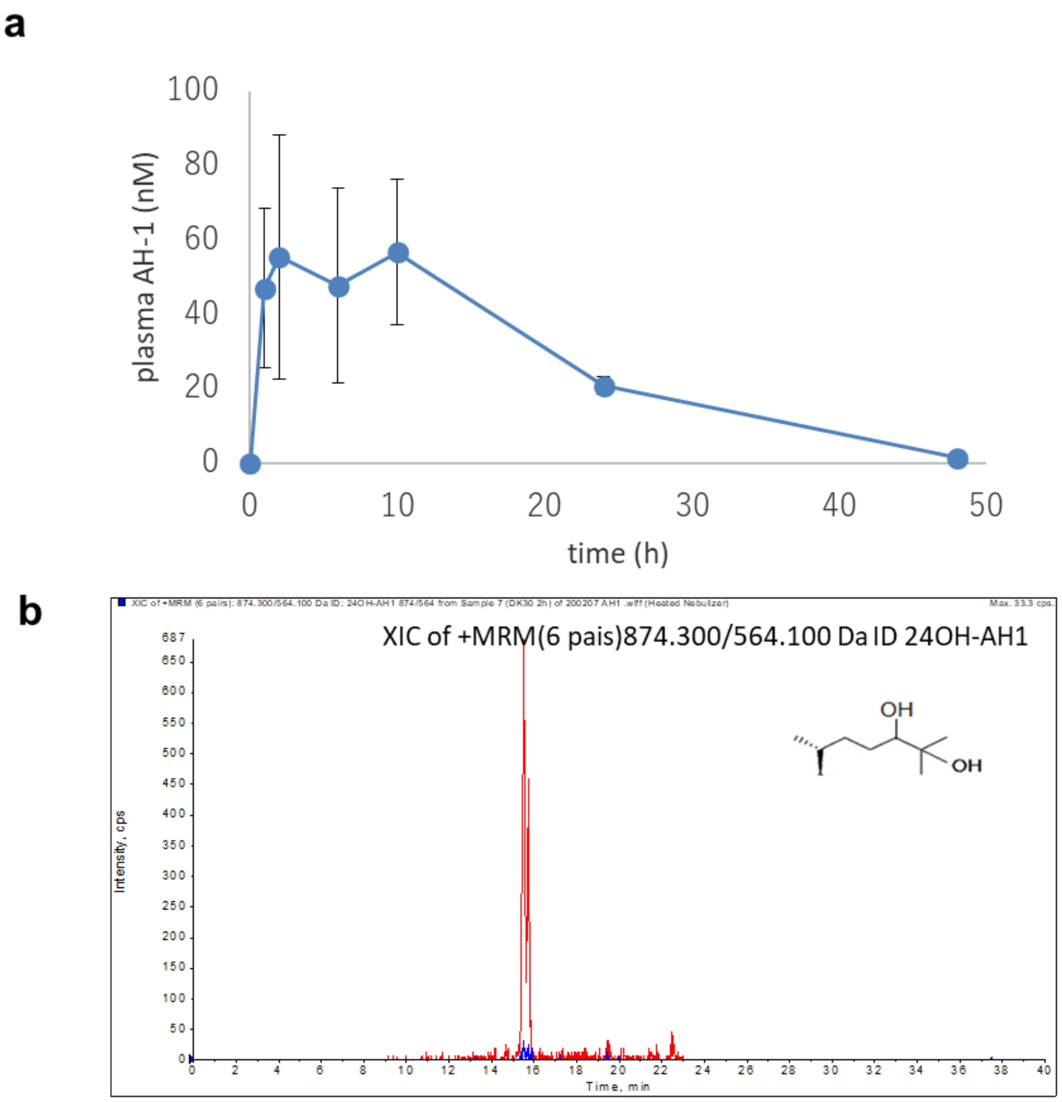

Supplemental Figure 2 Plasma clearance of AH-1 after single dosing.

(a) Time course of plasma concentration of AH-1 after single dosing of AH-1 (50µg/kg). Values of AH-1concentration are the means ± SEM (n=5 animals/group).

(b) MRM chromatogram of C24-hydroxy metabolite of AH-1 (24OH-AH-1) in Vdr(R270L) rat plasma after 24h from single dosing of AH-1 (50µg/kg).
